# Rapamycin impairs endothelial cell function in human internal thoracic arteries

**DOI:** 10.1186/s40001-015-0150-4

**Published:** 2015-06-24

**Authors:** David C. Reineke, Else Müller-Schweinitzer, Bernhard Winkler, Donatina Kunz, Moritz A. Konerding, Thomas Grussenmeyer, Thierry P. Carrel, Friedrich S. Eckstein, Martin T.R. Grapow

**Affiliations:** Department of Cardiovascular Surgery, University Hospital Berne, Bern, CH-3010 Switzerland; Department of Cardiac Surgery, University Hospital Basel, Spitalstrasse 21, Basel, CH-4031 Switzerland; Department of Biomedicine, University Basel, Basel, CH-4031 Switzerland; Department of Anatomy, Johannes Gutenberg-University, Mainz, 55099 Germany

**Keywords:** Internal thoracic artery, Rapamycin, Endothelial cells, Mammalian target of rapamycin, eNOS

## Abstract

**Background:**

Definitive fate of the coronary endothelium after implantation of a drug-eluting stent remains unclear, but evidence has accumulated that treatment with rapamycin-eluting stents impairs endothelial function in human coronary arteries. The aim of our study was to demonstrate this phenomenon on functional, morphological and biochemical level in human internal thoracic arteries (ITA) serving as coronary artery model.

**Methods:**

After exposure to rapamycin for 20 h, functional activity of ITA rings was investigated using the organ bath technique. Morphological analysis was performed by scanning electron microscopy and evaluated by two independent observers in blinded fashion. For measurement of endothelial nitric oxide synthase (eNOS) release, mammalian target of rapamycin (mTOR) and protein kinase B (PKB) (Akt) activation, Western blotting on human mammary epithelial cells-1 and on ITA homogenates was performed.

**Results:**

Comparison of the acetylcholine-induced relaxation revealed a significant concentration-dependent decrease to 66 ± 7 % and 36 ± 7 % (mean ± SEM) after 20-h incubation with 1 and 10 μM rapamycin. Electron microscopic evaluation of the endothelial layer showed no differences between controls and samples exposed to 10 μM rapamycin. Western blots after 20-h incubation with rapamycin (10 nM–1 μM) revealed a significant and concentration-dependent reduction of p (Ser 1177)-eNOS (down to 38 ± 8 %) in human mammary epithelial cells (Hmec)-1. Furthermore, 1 μM rapamycin significantly reduced activation of p (Ser2481)-mTOR (58 ± 11 %), p (Ser2481)-mTOR (23 ± 4 %) and p (Ser473)-Akt (38 ± 6 %) in ITA homogenates leaving Akt protein levels unchanged.

**Conclusions:**

The present data suggests that 20-h exposure of ITA rings to rapamycin reduces endothelium-mediated relaxation through down-regulation of Akt-phosphorylation via the mTOR signalling axis within the ITA tissue without injuring the endothelial cell layer.

## Background

The use of drug-eluting stents (DES) has markedly reduced the risk of restenosis after coronary angioplasty as compared to bare metal stents. Rapamycin or one of its derivates is the most widely employed drug in DES. It suppresses the growth of neo-intimal vascular smooth muscle cells by inhibiting the mammalian target of rapamycin (mTOR), a serine/threonine kinase that plays an important role in the regulation of cell proliferation [1–3]. However, with the frequency of late stent thrombosis increasing considerably after discontinuation of the dual antiplatelet therapy, concerns have recently arisen regarding adverse effects in some patients.

Early pharmacological experiments with rat aortic tissue comparing different immunosuppressive substances and their influence on endothelial function revealed beneficial effects of rapamycin. In contrast to cyclosporine-C and FK-506, both negatively influencing endothelial function, rapamycin even potentiated endothelium-dependent relaxation in these tissues [4, 5]. Furthermore, it has been shown in Lewis rats that besides preservation of endothelial function, sensitivity to vasospasm is not increased and normal levels of endothelial nitric oxide synthase (eNOS) protein expression are maintained [6].

However, in contrast to the observed reactions in rats, rapamycin depresses endothelium-dependent relaxation in pig coronary arteries in vitro [7]. These data are largely confirmed with paclitaxel-eluting stents. Paclitaxel is a mitotic inhibitor and was initially used as a chemotherapeutic agent. For the treatment of restenosis, its antiproliferative effect is of value by inhibiting neo-intimal growth within the coated stent resulting in scared tissue [8].

First functional data on human coronary arteries was reported by Togni showing that exercise-induced coronary vasomotion is totally different in segments proximal and distal to rapamycin and paclitaxel-eluting stents when evaluated 6 months after deployment [9]. While patients who had received bare-metal stents or reference vessels without stents revealed normal exercise-induced vasodilatation, patients with DES clearly demonstrated paradoxical vasoconstriction around the vessel segments of rapamycin and paclitaxel-eluting stents. By contrast, vasodilator response to nitroglycerin was maintained, suggesting a drug-induced endothelial dysfunction as the underlying mechanism [9, 10].

Our goal was, therefore, to investigate in a human artery model whether exposure of human internal thoracic arteries to rapamycin in vitro modifies vascular function and/or reduces nitric oxide production/release, thus studying directly the physiological and morphological influence of rapamycin on the human endothelium.

## Methods

### Organ bath experiments

**Fig. 1:**
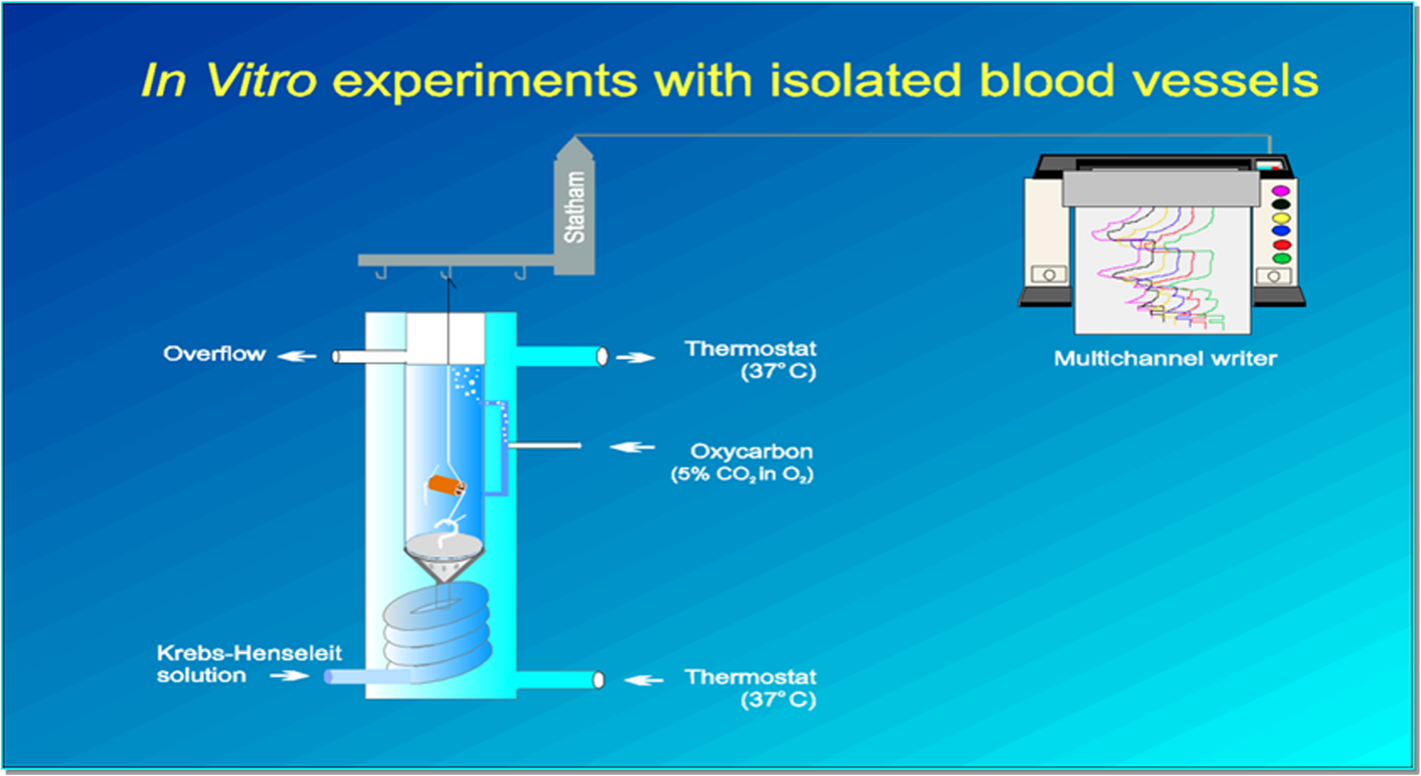
Schematic presentation of the experimental setup. Organ bath with the specimen containing KH solution at 37 °C and gassed continuously with 5 % CO_2_ in oxygen. Changes in the tone of the preparations are recorded with electromechanical transducers and a potentiometric recorder

Human internal thoracic arteries (ITA; 10–20 mm long, inner diameter 1–2 mm) were taken during bypass surgery at the beginning of the operation before induction of the cardiopulmonary circuit and after obtaining approval from the local ethical committee with patients’ informed consent. The ethics committee Basel county of the University of Basel Switzerland (Spitalstrasse 12, 4031 Basel, Basel, Switzerland) approved the study prior to conduction, and all patients were interviewed prior to the operation and had to give their consent in written form. Arteries from a total of 21 patients (19 males, 2 females) with a mean age of 64 years (range 56–84) were employed. Comprehensive patients’ details are presented in Table 1. The preparations were placed into RPMI culture medium or modified Krebs-Henseleit (KH) solution (composition mM: NaCl 118, KCl 4.7, MgSO_4_ 1.2, CaCl_2_ 1.25, KH_2_PO_4_ 1.2, NaHCO_3_ 25, glucose 11, EDTA 0.03) at room temperature and directly transported to the laboratory. The vessels were cleaned from loose connective tissue avoiding any traction or tension on the material using a microscope and microvascular instruments, cut into rings (about 2–3 mm long), mounted between two hooks of stainless steel wire (diameter 0.5 mm), suspended in 10-ml organ baths containing KH solution at 37 °C and gassed continuously with 5 % CO_2_ in oxygen (Fig. 1). Changes in the tone of the preparations were recorded isometrically under a resting tension of 1 g with electromechanical transducers (Statham model UC 3, Gould Inc., Oxnard, CA, USA) and a potentiometric recorder (Servorec 460, Morawtz Inc., Germany). At the beginning of the experiments, the rings were stretched to an initial tension of about 1.5 g and allowed to relax and equilibrate for about 2–3 h in the bathing medium. During this time, the baseline tension of the rings was readjusted to 1 g if required.

**Table 1:** Clinical profile, risk factors and preoperative therapy of the patients

	Organ bath on ITA rings	Western blot on ITA homogenates			
	NA and Ach	m-TOR	p-mTOR	Akt	p-Akt
Age (years)	64 (56–84)	62 (56–68)	64 (56–71)	68 (54–81)	68 (54–81)
Number of patients (*n*)	21	6	6	19	24
	*n* (%)	*n* (%)	*n* (%)	*n* (%)	*n* (%)
Smokers	6 (29)	2 (33)	2 (33)	5 (26)	9 (38)
Hypercholesterinemia	13 (62)	2 (33)	2 (33)	11 (58)	17 (71)
Arterial hypertension	11 (52)	4 (67)	5 (83)	14 (74)	16 (67)
Diabetes mellitus	2 (10)	0	1 (17)	9 (47)	9 (38)
Aspirin	12 (57)	5 (83)	6 (100)	17 (89)	20 (83)
Statine	14 (67)	6 (100)	6 (100)	15 (79)	21 (88)
Beta-blocker	14 (67)	4 (67)	4 (67)	15 (79)	18 (75)
ACE inhibitors	4 (19)	2 (33)	2 (33)	12 (63)	16 (67)
Calcium entry blockers	1 (5)	0	1 (17)	3 (16)	7 (29)

Thereafter, the rings were contracted with noradrenaline (NA; 1 μM). When the NA effect was levelling off, endothelial function was tested by adding acetylcholine (ACh; 1 μM). Only rings showing a minimum of 40 % ACh-induced relaxation compared to the maximum contractile response to NA were included in the study. After an equilibration period of 1 h, the experiment started by repeating the NA and ACh administration. After 1-h washout, the rings were incubated for 20 h in KH solution at 37 °C with rapamycin 0.1, 1, and 10 μM and ethanol in its highest concentration as solvent control. In each experiment, at least one ring served as a time-matched control preparation to correct for any sensitivity change. After 20 h, the rings were washed out, the preload was readjusted to 1 g during a further equilibration period of 1 h before NA and ACh were tested again.

### Scanning electron microscopy

For scanning electron microscopy, 12 arterial rings from four patients were carefully removed from the organ holders after the in vitro experiments, fixed at 4 °C for 24 h in 2.5 % phosphate-buffered saline (PBS)-buffered glutaraldehyde (pH 7.4, 300 mOsmol) and stored at 4 ° C in PBS buffer. The rings were cut longitudinally with a razor blade, dehydrated and critical-point dried with liquid CO_2_. The specimens were mounted on stubs with conductive silver paste, sputtered with 20 A gold in argon atmosphere and examined in a Philips ESEM XL 30 scanning electron microscope at 20 kV (kilovolt). Examination was performed by two independent observers in blinded fashion.

### Western blotting on ITA homogenates

Samples of the ITA were splitted equally and incubated in a six-well plate in RPMI containing 1 μM rapamycin or solvent as control for 20 h. Before protein extraction for Western blotting, the ITA segments were snap-frozen in liquid nitrogen and stored at −75 °C. The frozen ITA rings were homogenized with a mortar, and total protein was dissolved in lysis buffer (Ripa Sigma) with protease and phosphatase inhibitors (Roche CPI). Cell debris was removed by centrifugation, and supernatants were quantified by the protein assay, Precision Plus Protein Standards (BioRad). Thereafter, 35 myg of total protein was electrophoresed on two similar 10 % SDS-polyacryl-amide gels and immunoblotted in a tank blot onto polyvinylidene difluoride membranes (Life Technologies, TDE, Zug, CH). The membranes were probed either with anti-Akt primary antibody (1:500) or with anti-phospho-Akt Ser473 (1:500, Cell Signalling Technology) for 2 h at room temperature and then exposed to horseradish peroxidase (HRP)-conjugated anti-rabbit IgG (1:2000, Cell Signaling Technology). Probing against mTOR was done either with a c-terminus-specific antibody (1:500, Sigma) or with a phospho-Ser2481-specific antibody (1:1000, Cell signaling) overnight at 4 °C. For standardization, glyceraldehyde 3-phosphate dehydrogenase (GAPDH), detected with a monoclonal antibody (1:1000, Sigma), was employed. For chemiluminescence, a commercially available ECL-Substrate (Pierce, “SuperSignal® West Pico Chemiluminescent Substrat”) was used. Band density was measured with Quantity One (BioRad) software and normalized to the GAPDH band in the same lane by dividing the densities by the GAPDH band density. Densitometric quantification was performed as described above.

### Western blotting on Hmec-1

Hmec-1 were cultivated in MCDB131 (Invitrogen) containing 2 % FCS, 0.2 μg/ml hydrocortisone (Sigma), 10 ng/ml EGF (Sigma) and 100 μM ascorbic acid (Sigma). Cell and tissue extraction was performed in RIPA buffer (Sigma R0278) including 1 % protease inhibitor cocktail (Sigma P8340), 1 % phosphatase inhibitor cocktail 3 (Sigma P0044), 1 mM Na-orthovanadate and 10 mM pyrophosphate. Thereafter, 30 μg protein of cell lysates was separated by SDS-PAGE (7 % acrylamide gels) and electrophoretically transferred to polyvinylidene fluoride (PVDF) membranes (17 h, 150 mA). After transfer, membranes were blocked with a caseine-based blocking buffer (Sigma, B6429). For probing against protein phosphorylation on Ser1177 (p-eNOS), we used 1:1000 diluted SAB4300128 (Sigma) for 3 h at room temperature. Antibodies against GAPDH (glycerinaldehyd-3-phosphat-dehydrogenase, Sigma, G9545) were used. For reaction with a second antibody, peroxidase-conjugated anti-rabbit antibodies (Sigma, A0545) were used. For visualization of peroxidase signals, an enhanced luminescence protocol based on 1.25 mM luminol (Sigma, A4685) in 100 mM Tris, pH 8.5, 0.6 mM p-coumaric acid (Sigma, C9008) and 0.008 % hydrogen peroxide (Sigma 216763,) was used. Densitometric quantification was performed with “ImageJ” (ImageJ, java based, NIH, MA, USA). In case of strong and inhomogeneous background, first profiles for each lane with Image J were generated. Profile raw data were exported to Excel to determine and calculate net peak areas. For normalization of protein quantifications, the GAPDH signal which was generated in a second probing step on the same membranes was used.

### Drugs used for functional studies

The following drugs were used: (−)-Noradrenaline hydrogen tartrate, rapamycin (Sirolimus), (Fluka, Buchs, Switzerland), and acetylcholine chloride (Dispersa A.G., Hettlingen, Switzerland).

Rapamycin was dissolved in ethanol and diluted in distilled water to give 1 ml solution/60 % ethanol.

### Data analysis

Data analysis and graphic illustrations were made with Origin software (Microcal Software Inc., Northampton, MA, USA). Where appropriate, one-way analysis of variance (ANOVA) was performed, followed by the Bonferroni-corrected *t* test to assign differences to individual between-group comparisons when overall significance (*P* < 0.05) was attained. Data are presented as mean values ± SEM.

## Results and discussion

### Functional activity

**Fig. 2:**
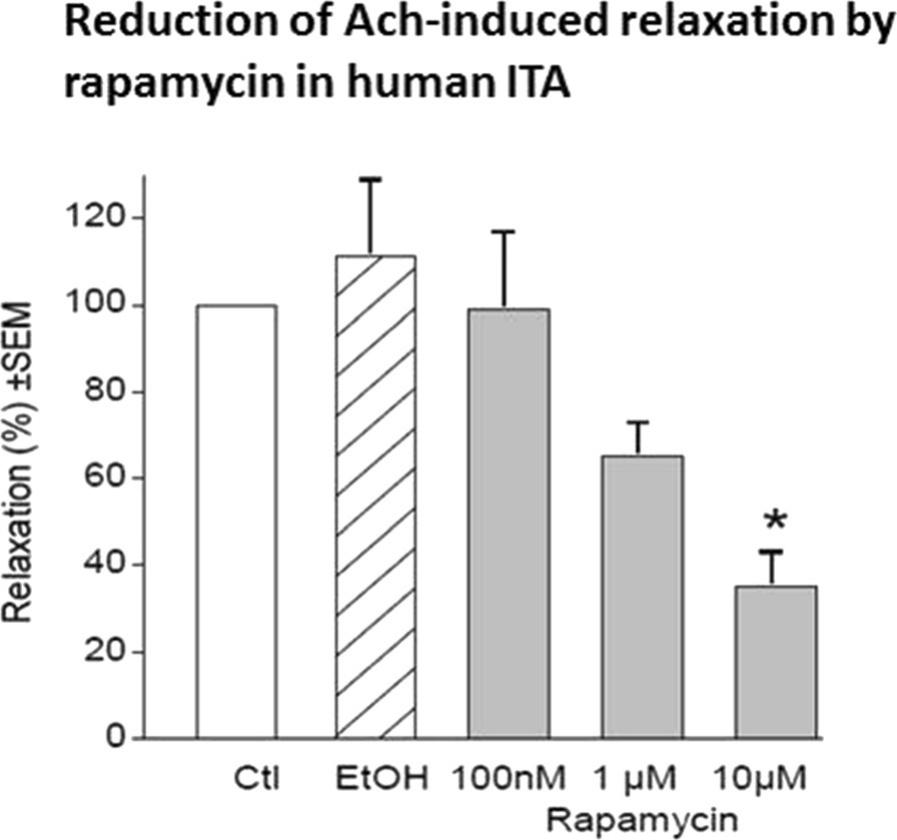
Relaxation of noradrenaline-precontracted ITA rings to acetylcholine after exposure for 20 h to rapamycin (*grey columns*), EtOH solvent (*hatched column*) and in time-matched controls (*empty column*). *Asterisk* indicates significant difference to EtOH exposure. For each column *n* = 8–10, *bars* represent SEM

Preceding studies revealed that neither contractile responses to NA nor relaxant activity of acetylcholine (ACh) was modified, when ITA rings had been exposed for 20 min to rapamycin (10 nM–1 μM, not illustrated). Incubation for 20 h of ITA rings with rapamycin again did not modify contractile responses to NA (1 μM); however, endothelium-dependent relaxant responses to ACh were significantly attenuated in a concentration-dependent manner when ITA rings had been exposed for 20 h to rapamycin (Fig. 2).

### Scanning electron microscopy

**Fig. 3:**
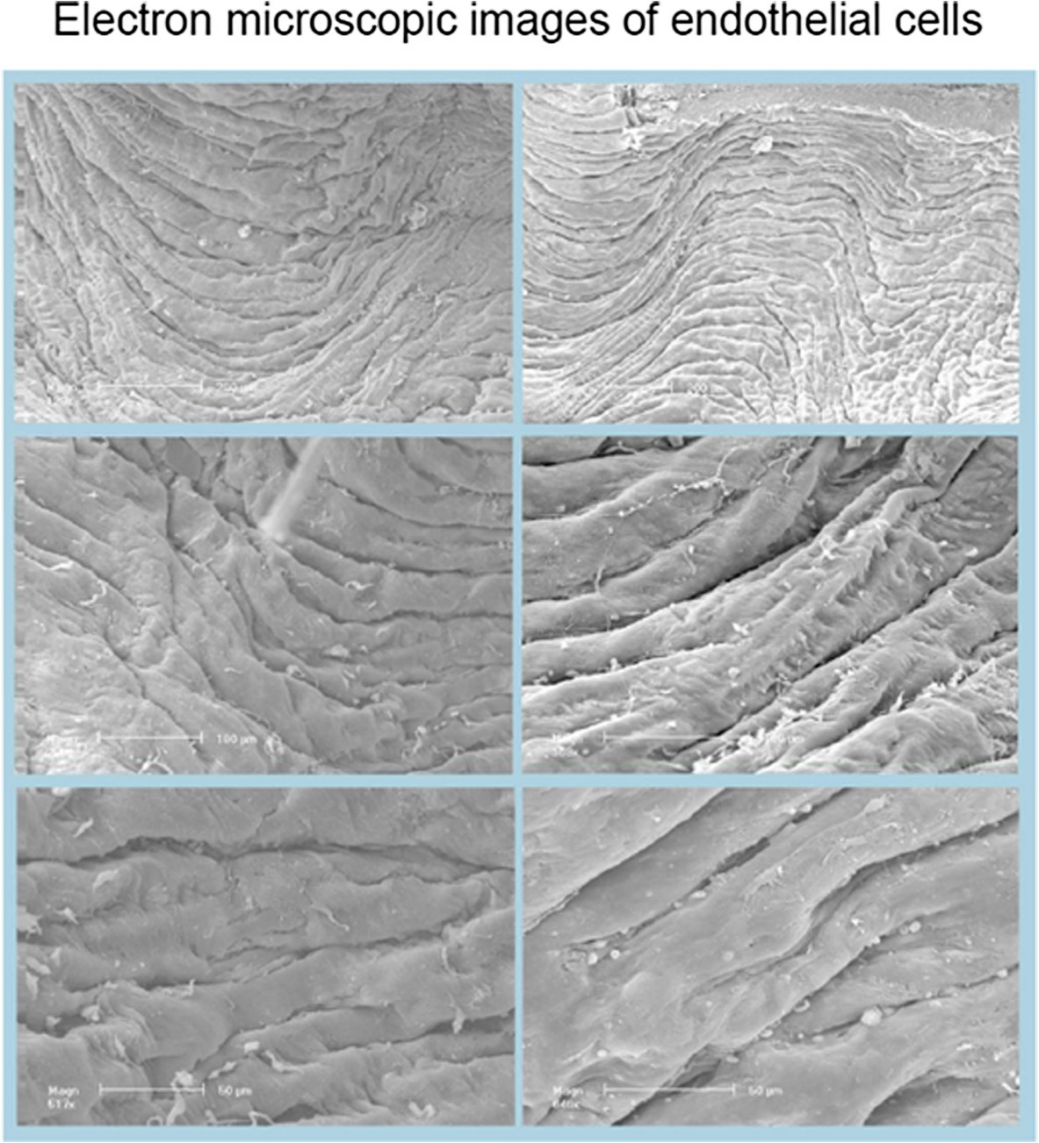
Representative scanning electron micrographs of the endothelium of human internal arteries after exposure to solvent (*left*) and after exposure to 10 μM rapamycin (*right*) for 20 h taken from the same patient. Different magnifications are indicated by *bars* of 200, 100 and 50 μm (*from top to bottom*). Some depositions of blood cells and noncellular material are seen in both groups. However, the endothelial layer is well preserved in both groups, i.e. there were no differences between rapamycin-exposed and control rings

Following 20-h exposure to rapamycin, the endothelial layer was well preserved. There were no significant differences between rapamycin-exposed and control rings. Some spikes and blebs indicating cell cytoskeleton damage could be noticed in both groups, but with the exception of a few spots, the integrity of the layer was complete and intact (Fig. 3).

### mTOR, Akt and eNOS phosphorylation in ITA homogenates

**Fig. 4:**
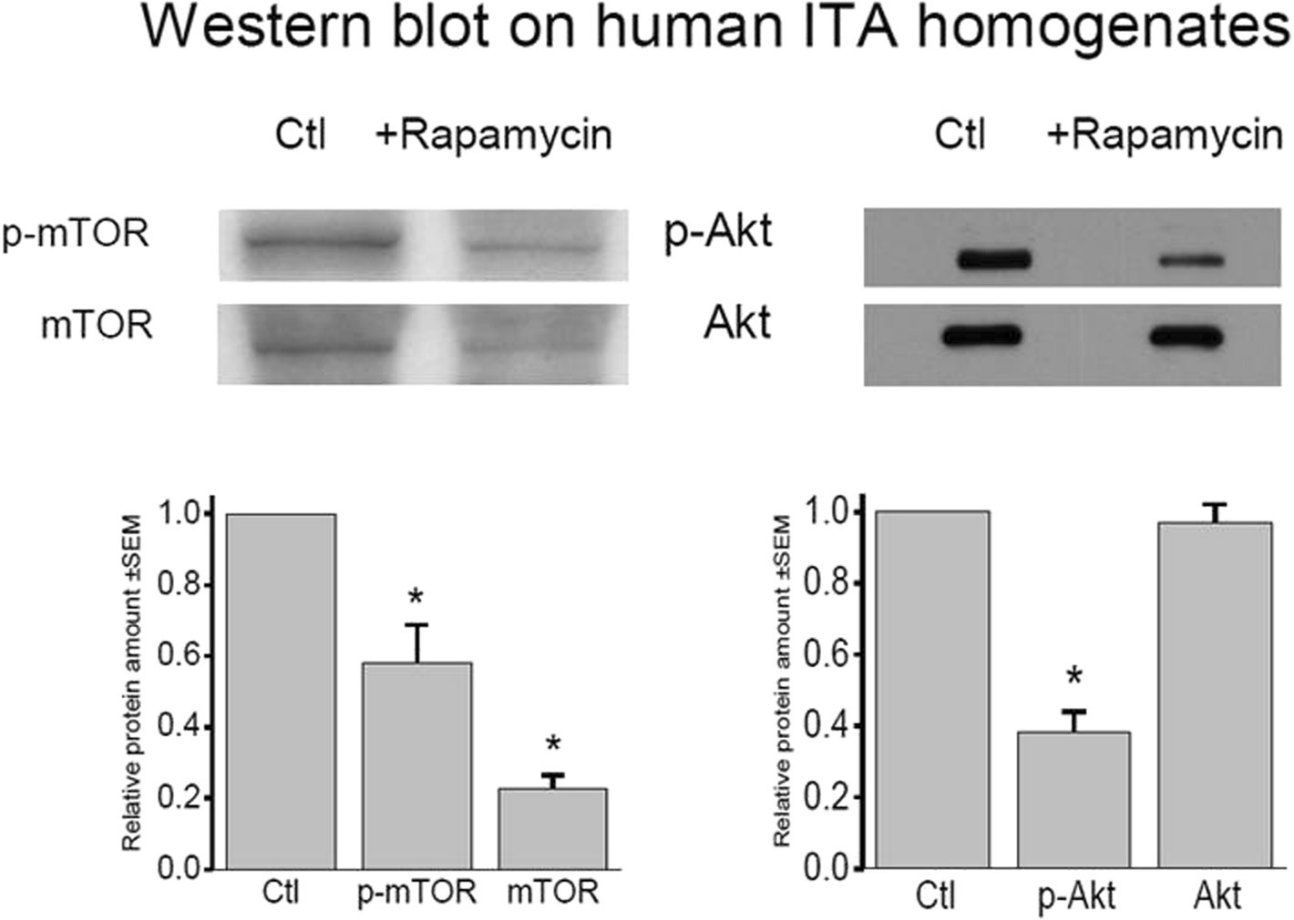
Left-hand traces. *Top*: Representative Western blot bands for phospho (Ser 2481)-mTOR (p-mTOR) and mTOR (anti-c-terminal antibody) on internal thoracic artery tissue. *Bottom*: *Columns* indicate repression of p-mTOR and mTOR on ITA tissue exposed for 20 h to 1 μM rapamycin in relation to controls (*Ctl*). *Asterisk* indicates statistical significance for down-regulation. Each test was performed and normalized in six experiments, and the *bars* represent SEM. Right-hand traces. *Top*: Representative Western blot bands for phospho (Ser 473)-Akt (p-Akt) and Akt on internal thoracic artery tissue. *Bottom*: *Columns* indicate changes of p-Akt (*n* = 24) and Akt (*n* = 19) in ITA tissue exposed for 20 h to 1 μM rapamycin in relation to controls (*Ctl*). *Asterisk* indicates statistical significance for down-regulation, and the *bars* represent SEM

When the effect of rapamycin was tested in homogenates from ITA segments, a significant (*p* < 0.001) reduction of the protein phosphorylation on Ser473 (p-Akt) indicated substantial down-regulation of Akt phosphorylation, whereas Akt protein levels remained unchanged (*p* = 0.59, Fig. 4, right).

To investigate whether the signalling axis mTOR-Rictor is involved in down-regulation of Akt-phosphorylation, mTOR-phosphorylation and mTOR protein levels after rapamycin treatment were analysed by Western blotting. We focussed on Ser 2481 phosphorylation because this site was recently reported to monitor mTOR signalling towards Rictor-specific targets [11]. There was a significant reduction of the phospho-form of mTOR in extracts of in vitro-treated ITA samples by 42.0 ± 10.8 % (*p* < 0.01, *n* = 6) and a reduction by 77.3 ± 3.7 % (*p* < 0.005 *n* = 6) of mTOR protein compared to corresponding controls (Fig. 4, left).

### eNOS phosphorylation Hmec-1

**Fig. 5:**
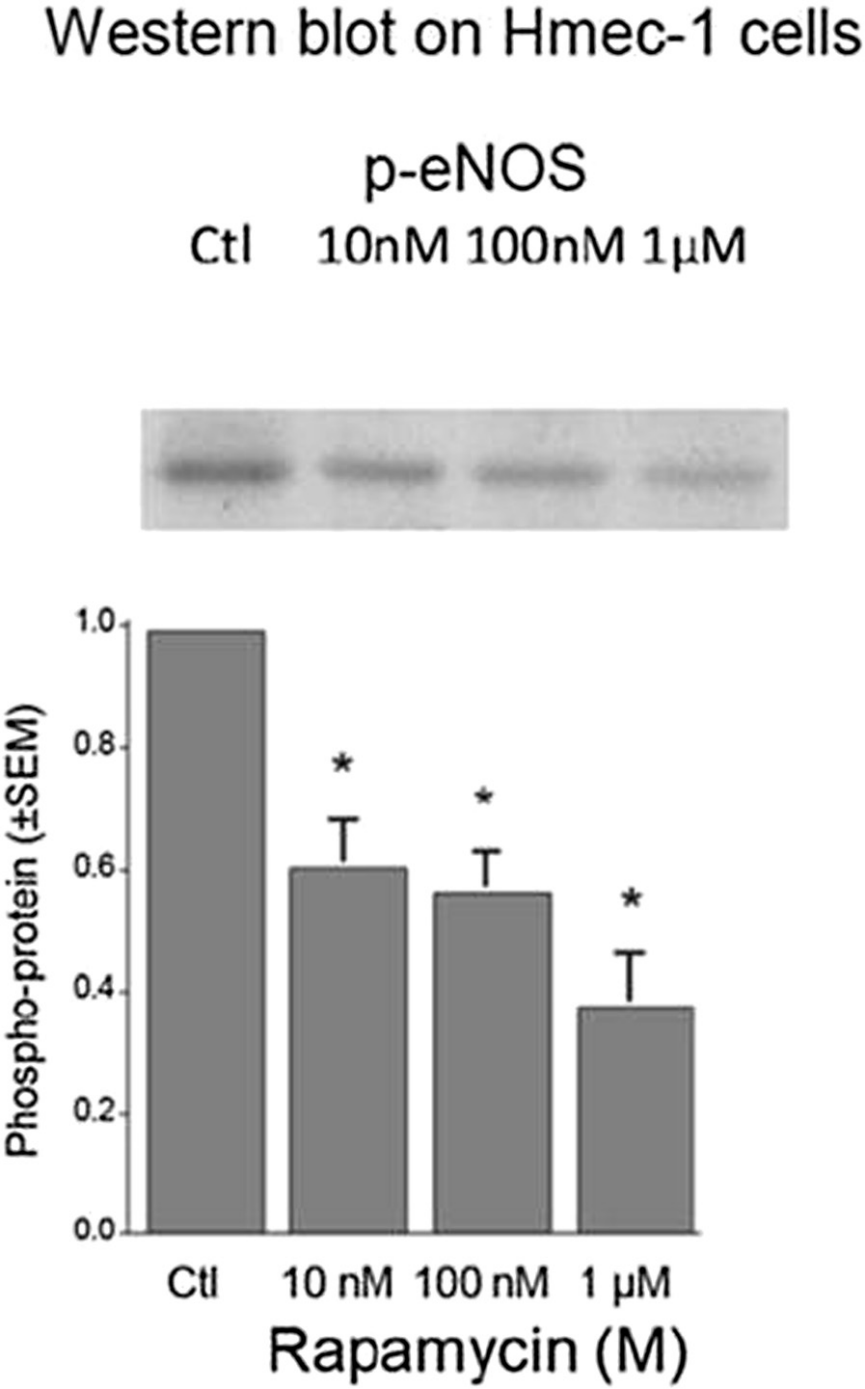
*Top*: Representative Western blot bands for phospho (Ser 1177)-eNOS (p-eNOS) in Hmec-1 after exposure to different concentrations of rapamycin for 20 h in relation to controls (*Ctl*). *Bottom*: The *columns* indicate activation of phospho-protein by eNOS after exposure to different concentrations of rapamycin for 20 h in relation to controls (*Ctl*). Each concentration was tested and normalized in five to six experiments, and the *bars* represent SEM. *Asterisk* indicates significance against controls

Because of the low fraction of endothelial cells in human arteries, the influence of rapamycin on eNOS activation was investigated in the human endothelial cell line Hmec-1. After 20-h incubation with rapamycin, a concentration-dependent reduction of the phosphorylation on Ser1177 (p-eNOS) by 38.7 % (±6.8 % (SEM), *p* = 0.00116, *n* = 6; 10^−8^ M rapamycin), 42.8 % (±5.7 % (SEM), *p* = 0.00033, *n* = 6; 10^−7^ M rapamycin) and 68.5 % (±7.7 % (SEM), *p* = 0.00067, *n* = 5; 10^−6^ M rapamycin) indicating substantial down-regulation of eNOS activity was observed (Fig 5).

## Conclusions

Since sirolimus also known as rapamycin was discovered as a product of the bacterium *Streptomyces hygroscopicus*, it is used worldwide. The first sirolimus-eluting coronary stent received CE Mark approval in Europe in April 2002. Stent-induced restenosis involves a complex interaction of biological events, endothelial injury and injury due to lacerations of the arterial wall. Sirolimus is known to have severe systemic adverse effects like cancerogenesis or lung toxicity. When used to prevent restenosis, these concerning side effects should not be left totally unconsidered.

Our aim was therefore to investigate in a human artery setting if exposure of human internal thoracic arteries to rapamycin in vitro modifies vascular function or nitric oxide production, thus studying directly the physiological and morphological influence of rapamycin on the human endothelium.

The main finding of the present study is that incubation of human ITA in vitro for 20 h with rapamycin significantly attenuated the endothelium-dependent relaxation in response to ACh in a concentration-dependent manner. At the same time, Western blots of rapamycin-treated human endothelial cell lines revealed a significant concentration-dependent reduction of eNOS release. In addition, Western blots of similarly incubated ITA homogenates showed a significantly reduced activation of mTOR, p-mTOR and p-Akt while leaving Akt protein levels unchanged, thus supporting the functional data.

The concentrations used in the present study are on the low end compared to concentrations attained in vivo, and it is likely that significantly higher local drug concentrations are attained in arteries located in close proximity to a drug-eluting stent because the lipophilicity of rapamycin considerably enhances the uptake by the arterial wall [12, 13]. Especially if serial stenting is applied or multiple stents are used within the same coronary system, e.g. left main stem, drug concentrations may vary and reach significantly higher peak concentrations in the downstream vessel. The present data thus confirms clearly in vivo results from major clinical studies showing impaired endothelium-dependent relaxations in response to exercise or acetylcholine application in coronary arteries distal to drug-eluting stents pointing to a cumulative downstream watershed impact of an upstream-eluted drug compound. Patients with bare-metal stents are not affected [10, 14]. However, in contrast to the functional effect of rapamycin in the present study, electron microscopic examinations showed no short-term effect of rapamycin exposure on vessel wall morphology.

The mid-term and long-term effect of endothelial renewal, cell migration and cell composition under the influence of rapamycin is currently in focus of several studies, and it is already known that mTOR regulates vascular smooth muscle cell differentiation and plays an important role in directing cell faith and endothelial composition after intervention or vascular injury. With mTOR being a central element in signalling pathways involved in the control of cell growth and proliferation, rapamycin also seems to affect the number, homing and proliferation of endothelial progenitor cells resulting in the prevention of endothelial healing [11–17].

Reduction of eNOS mRNA and protein expression by rapamycin have been reported by several groups [18, 19]. Treatment of cultured human umbilical vein endothelial cells with rapamycin increases Akt activity after brief (up to 1 h) exposure while longer exposure (more than 4 h) decreases Akt phosphorylation and activity, as determined by phosphorylation of its substrate glycogen synthase kinase-3.

Protein-kinase B (PKB/Akt) normally mediates activation of eNOS, leading to increased NO production. However, rapamycin inhibits PKB/Akt via binding to mTOR resulting in decreased NO production [20, 21]. In the present experiments, a significant reduction of the protein phosphorylation of p-mTOR and p-Akt in human ITA homogenates is in line with the functional data in ITA rings suggesting that the rapamycin effect might occur via the mTOR/Rictor pathway. Because of the low fraction of endothelial cells in human ITA, the influence of rapamycin on eNOS activation was investigated in the human microvascular endothelial cell line Hmec-1. Accordingly, after a 20-h incubation period of Hmec-1 with rapamycin, a significantly reduced concentration-dependent eNOS phosphorylation was found.

These mechanisms explain the present observations that rapamycin attenuated both function and NO production/release by endothelial cells of ITA rings from a representative population in vitro although scanning electron microscopy did not show any acute structural attenuation of the integrity of the endothelial layer between the groups at an early stage.

The most important stimulus for expanding the coronary flow reserve is shear stress generated by streaming blood along the endothelial layer. Thereby, continuous formation of NO with a tremendous effect on coronary vasodilation is induced. Shear stress stimulates phosphatidylinositol-3-OH-kinase (PIxK), activating Akt/PKB by phosphorylation, which then phosphorylates eNOS protein, leading to an increased sensitivity to calcium. If so, it is already active in producing NO at sub-physiological calcium levels [22–24].

However, in the presence of rapamycin, vascular endothelial growth factor, tumor necrosis factor and insulin (like shear stress) failed to phosphorylate Akt/PKB, supporting the contention that mTOR regulates Akt/PKB activation in endothelial cells as reported .

In summary, we have demonstrated that incubation of ITA in vitro for 20 h with rapamycin did not damage endothelial cells histologically and that rapamycin attenuated both endothelial function in ITA rings and activation of eNOS in human endothelial cell lines in vitro. Furthermore, it was found that exposure of ITA homogenates to rapamycin reduced activation of mTOR and p-Akt suggesting that rapamycin inhibited eNOS in endothelial cells in ITA via the mTOR/Rictor pathway.

### Limitations

The current study can only mimic the pathophysiological mechanisms leading to impaired coronary artery function in a clinical setting after drug-eluting stent implantation. The present experiments were performed on ITA serving as an established human artery model. The results are extrapolated to human coronary arteries where the effects of rapamycin are obvious. As opposed to the majority of existing data from animal studies, these experiments were performed on human material again emphasizing that marked species differences do exist.

## Abbreviations

ACh: acetylcholine
Akt: protein kinase B (PKB)
Ctl: controls
DES: drug-eluting stents
eNOS: endothelial NO synthase
EtOH: ethanol
FCS: fetal calf serum
FK-506: Tacrolimus®
GAPDH: glyceraldehyde 3-phosphate dehydrogenase
ITA: internal thoracic arteries
KH: Krebs-Henseleit
mTOR: mammalian target of rapamycin
NA: noradrenaline
